# Potential benefits of precise corticosteroids therapy for severe 2019-nCoV pneumonia

**DOI:** 10.1038/s41392-020-0127-9

**Published:** 2020-02-21

**Authors:** Wei Zhou, Yisi Liu, Dongdong Tian, Cheng Wang, Sa Wang, Jing Cheng, Ming Hu, Minghao Fang, Yue Gao

**Affiliations:** 1 Department of Pharmaceutical Sciences, Beijing Institute of Radiation Medicine, 100850 Beijing, P. R. China; 2 School of Nursing, Capital Medical University, 100069 Beijing, P. R. China; 3 Department of Pharmacy, Hebei General Hospital, 050000 Shijiazhuang, P. R. China; 4 Department of Orthopedics, First Medical Center, General Hospital of Chinese PLA, 100853 Beijing, P. R. China; 5 Emergency Department, The Second Affiliated Hospital of Zhejiang University School of Medicine, 310009 Hangzhou, P. R. China; 6 Emergency Surgery Department, Tongji Hospital Affiliated to Tongji Medical College, Huazhong University of Science and Technology, 430030 Wuhan, P. R. China; 7 Intensive Care Unit, Wuhan Pulmonary Hospital, 430030 Wuhan, P. R. China; 8 Emergency and Intensive Care Unit, Tongji Hospital Affiliated to Tongji Medical College, Huazhong University of Science and Technology, 430030 Wuhan, P. R. China

**Keywords:** Infectious diseases, Respiratory tract diseases

**Dear Editor**,

Since last December, the outbreak of 2019-nCoV in Wuhan has caused ever-increasing attention and public panic all over the world. Up to February 9, 2020, 40,171 patients had been diagnosed with 2019-nCoV infection, including 6484 (16.14%) severe cases and 908 deaths (2.27%). Compared to SARS and MERS, 2019-nCoV appears to be much more contagious but less lethal, as most patients have mild symptoms and good prognosis^1,2^. However, according to the Chinese government’s daily report, 13.2–21.3% of patients with 2019-nCoV infection developed into severe or fatal illness (Fig. S1a), which is characterized by the rapid development to acute respiratory distress syndrome (ARDS) or septic shock. Along with an increasing number of confirmed cases, the cumulative total of severe patients with 2019-nCoV is growing (Fig. S1b). Treatment of these critically ill patients is becoming one of the major challenges we are facing.

Unfortunately, there are still no specific antiviral medicines or vaccines recommended for 2019-nCoV infection. For patients with severe clinical manifestations, an effective clinical treatment scheme is of great importance. On February 7, 2020, the China’s National Health Commission released the fifth trial version of Diagnosis and Treatment Scheme for Pneumonitis with 2019-nCoV Infection, and provided a systematic treatment strategy for severe cases. Remarkably, systematic corticosteroids treatment (methylprednisolone, <1–2 mg per kg body weight, for 3–5 days) was recommended to be an adjuvant therapy^3^, which immediately raised concerns about whether patients infected with this novel coronavirus could benefit from corticosteroids therapy ^4^.

## Salvage corticosteroids treatment for critical patients with 2019-nCoV?

Corticosteroids are widely used to prevent lung injury caused by severe community-acquired pneumonia (sCAP) due to their excellent pharmacological effects on the suppression of exuberant and dysfunctional systematic inflammation^5^. Some scholars may not support the corticosteroids treatment for novel coronavirus pneumonia (NCP), because observational studies and systematic reviews have indicated inconclusive clinical evidence on the effect of corticosteroids therapy for viral pneumonias (such as SARS, MERS and H1N1). Additionally, pulse-dose therapy or long-term administration to high dose of corticosteroids in early stage were reported to be possibly harmful^6–8^. However, these conclusions obscured the clinical benefits of corticosteroids on some subgroups of patients, particularly those with severe symptoms, as the clinical effects might be related to the indication (severities of illness), the timing of intervention, the dose and duration of corticosteroids therapy^9^.

Of note, as documented in a series of randomized clinical trials (RCT), low or physiologic dose of corticosteroids treatment did not reduce mortality from septic shock caused by primary lung infections, but it could bring clinical benefits to secondary outcomes, such as earlier reversal of shock, shorter duration to exit from ICU and mechanical ventilation^9,10^. Besides, salvage corticosteroids treatment for severe patients with advanced ARDS could alleviate the pulmonary fibrosis and prevent progressive pathological deterioration^11^, which provides a good framework for explaining why some critical patients with SARS infection benefit from rescue corticosteroids therapy. More importantly, mortality benefit favored the severe HIN1-illness in the adjunctive treatment group with low dose of corticosteroids^12^. Evidently, all these results strongly suggest that proper use of low-dose corticosteroids may bring survival advantages for critically ill patients with 2019-nCoV, but this treatment should be strictly performed on NCP patients with definite clinical indications (such as refractory ARDS, sepsis or septic shock) according to the recommended guidelines.

## Current evidence: clinical benefits of corticosteroids therapy for critical NCP patients

**Fig. 1:**
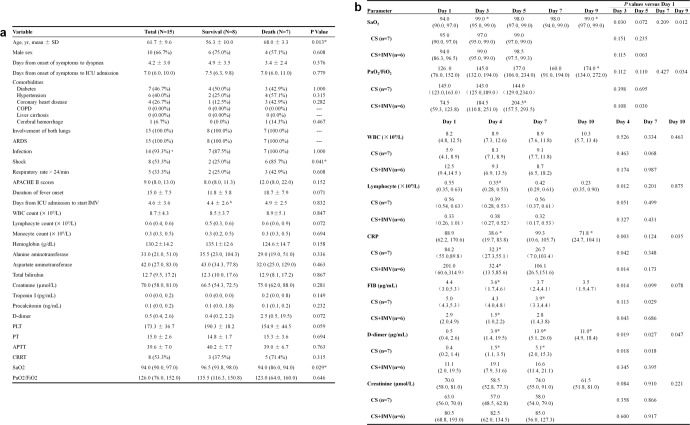
Corticosteroids treatment for severely ill patients with 2019-nCoV. **a** Demographics and baseline characteristics of patients infected with 2019-nCoV according to survival condition. ^a^Of the 14 infections, 12 with identified pathogens, 2 diagnosed by characteristic clinical symptoms without identified pathogens; ^b^N = 7. *P < 0.05, a significant difference between the two groups. **b** Part of indexes of 15 critically ill patients with 2019-nCoV infection during corticosteroids therapy. *P < 0.05 vs. the indexes on day 1. SaO_2_ oxygen saturation, CS corticosteroids, IMV invasive mechanical ventilation, PaO_2_/FiO_2_ ratio of arterial oxygen tension to fractional inspired oxygen concentrations, WBC white blood cell, CRP C-reaction protein, FIB fibrinogen

Over the past month, we collaborated with front-line ICU physicians and firstly evaluated the efficacy of corticosteroids treatment for severe or fatal cases with 2019-nCoV infection in Wuhan. From January 1 to January 29, 2020, the first 15 confirmed critical NCP patients with an average age of 61.7 years were admitted to the ICU in Wuhan Pulmonary Hospital. Of the 15 patients, 15 (100.0%) showed bilateral pneumonia, hypoxemia and moderate or severe ARDS, 14 (93.3%) had infections, 8 (53.3%) accompanied by shock and 9 (60.0%) with multiple organ injuries. All patients had received treatments containing noninvasive oxygen therapy and antibiotics and/or antiviral agents before and after ICU admission, and hypoxemia was not improved by these treatments. According to the guidelines, corticosteroids therapy (median hydrocortisone-equivalent dose of 400.0 mg/day) was instantly initiated after ICU admission for an average of 9.5 days, and outcomes for all patients were followed up until February 9, 2020 (Fig. 1a and Fig. S1c). Briefly, we observed that ICU mortality of these severe or fatal NCP patients was 46.7% (7/15), closer to that after adjustment for time-varying confounders induced by critically ill patients with MERS without corticosteroids treatment^6^, suggesting that corticosteroids might not improve ICU mortality in critical NCP patients. But meanwhile, systematic corticosteroids therapy in the first 3−5 days could enhance oxygen saturation (SaO_2_) and arterial oxygen tension (PaO_2_)/inspiratory oxygen fraction (FiO_2_), both of which could be further augmented by collaborating with invasive mechanical ventilation (IMV) (Fig. 1b). Corticosteroids did not exert any intervention efficacy on survival advantage of NCP patients complicated with both ARDS and shock or multiple organ injury (seven patients, all dead). Nevertheless, corticosteroids treatment in the phase of ARDS would effectively inhibit furious inflammatory storm (Fig. 1b) and gain valuable time for controlling infection and preventing secondary multiorgan damage and shock, which implies that corticosteroids have synergistic biological effects when combined with other intensivists’ treatment against severe or fatal NCP patients.

Due to the deficiency of sample size and a matched control group, we could not easily draw an accurate conclusion about the role of corticosteroids in patients with 2019-nCoV by now. However, our clinical experience and available descriptive data from the therapeutic process of the first 15 critical NCP patients are prone to support corticosteroids treatment for specific subgroup of critically ill patients with 2019-nCoV.

## Precautions of corticosteroids treatment in patients with 2019-nCoV

There is no fixed clinical guideline for the use of corticosteroids in critically ill patients in ICU. The anecdotal experience from SARS and sCAP therapy strongly supports precise corticosteroids management of NCP. Personalized medicine strategy should contain, but not limited to, specific indications, timing and duration, as well as therapeutic monitoring of corticosteroids therapy. As mentioned above, corticosteroids should be avoided unless there are indications for moderate or severe ARDS, sepsis or septic shock, in part consistent with the recommended clinical guidance from World Health Organization (WHO). We also do not suggest the use of corticosteroids for mild or early-stage ARDS, because early corticosteroids application could delay the clearance of virus and increase mortality risk, and corticosteroids are more likely to function on inflammation-mediated lung injury and interstitial fibro-proliferation at late-stage of ARDS^11^. Furthermore, clinical adverse complications in SARS patients with corticosteroids treatment have been reported to be dose-related. Over 240 mg of hydrocortisone-equivalent dose or an excessive cumulative dose was considered to be able to generate some side effects, including hyperglycemia, psychosis, and secondary infection, avascular necrosis^9,10^. Hence, lower dose and short duration of corticosteroids treatment (methylprednisolone, <1 mg/kg body weight, no more than 7 days), along with adverse drug reaction monitoring, would be more beneficial in clinical management of critical patients with 2019-nCoV. In addition, a long-term follow-up (6 months to 3 years) is essential to identify delayed adverse effects in these patients. Of course, the optimal treatment strategy requires constant adjustment as patient’s clinical performance changes.

In conclusion, Chinese government has taken effective measures to prevent a possible national or worldwide 2019-nCoV pandemic. Offering the most reasonable treatment to severe NCP patients could be another challenge we will face. We endorse the potential benefits from low-dose corticosteroids treatment in a subset of critically ill patients with 2019-nCoV based on existing studies and clinical experience, despite there is no significant improvement in overall survival. Certainly, our ongoing well-designed prospective cohort study with sufficient samples may provide systematic answers to this clinical dilemma—“to use or not to use corticosteroids for the treatment of lung injury with 2019-nCoV”—in the near future.

## Supplementary information

Supplementary Material
