# Supplementary material for: Potential benefits of precise corticosteroids therapy for severe 2019-nCoV pneumonia
Source: Signal Transduct Target Ther. 2020 Feb 21;5:18. doi: 10.1038/s41392-020-0127-9 (PMC7035340; doi:10.1038/s41392-020-0127-9)
Supplement: Supplementary file 1 — Supplementary Material [file 41392_2020_127_MOESM1_ESM.docx]

Supplementary Materials for

**Potential Benefits of Precise Corticosteroids Therapy for Severe 2019-nCoV Pneumonia**

Wei Zhou^1, †^, Yisi Liu^2, †^, Dongdong Tian^3, †^, Cheng Wang^4, †^, Sa Wang^5^, Jing Cheng^6^, Ming Hu^7^, Minghao Fang^8^, Yue Gao^1^

Correspondence to: *Yue Gao (*[*gaoyue@bmi.ac.cn*](mailto:gaoyue@bmi.ac.cn)*) or Minghao Fang (*[*fangmh@tjh.tjmu.edu.cn*](mailto:fangmh@tjh.tjmu.edu.cn)*) or Ming Hu (*[*huming74@163.com*](mailto:huming74@163.com)*).*

**This PDF file includes:**

Figure. S1

Figure. S1.


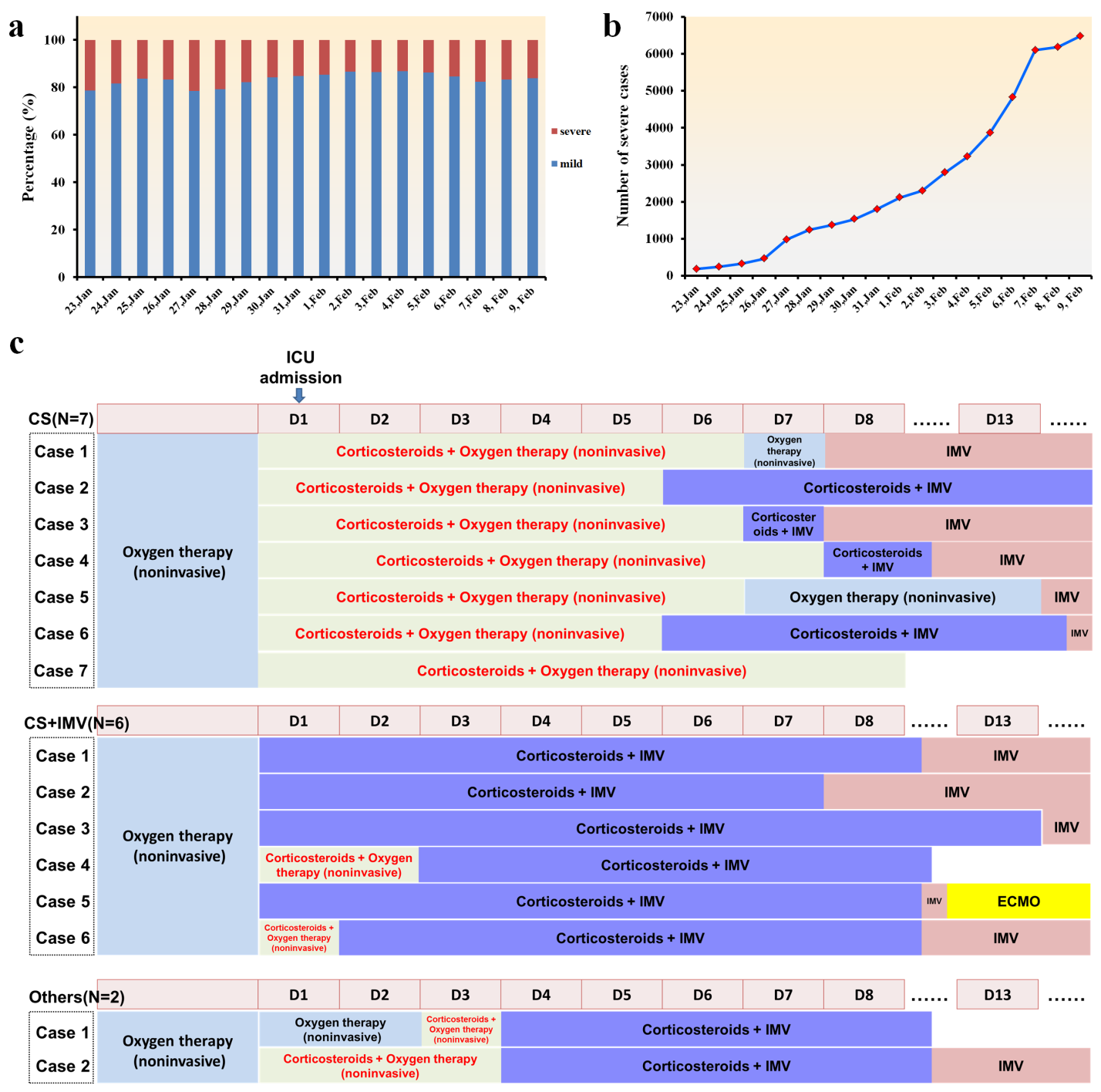


**Fig. S1** (**A**) The percentage and (**B**) the cumulative number of severe cases reported between Jan 23 to Feb 9, 2020, in China; (**C**) Schedules of corticosteroids treatment and IMV for the 15 NCP patients according to day of ICU admission. 15 patients had received treatments including oxygen therapy (non-IMV) and antibiotics and/or antiviral agents before ICU admission.
